# Lymphopenia is an important prognostic factor in peripheral T-cell lymphoma (NOS) treated with anthracycline-containing chemotherapy

**DOI:** 10.1186/1756-8722-4-34

**Published:** 2011-08-15

**Authors:** Yu Ri Kim, Jin Seok Kim, Soo Jeong Kim, Hyun Ae Jung, Seok Jin Kim, Won Seog Kim, Hye Won Lee, Hyeon Seok Eom, Seong Hyun Jeong, Joon Seong Park, June-Won Cheong, Yoo Hong Min

**Affiliations:** 1 Division of Hematology, Department of Internal Medicine, Yonsei University College of Medicine, Seoul, 120-752, Korea; 2 Division of Hematology/Oncology, Department of Medicine, Samsung Medical Center, Sungkyunkwan University School of Medicine, Seoul, 135-710, Korea; 3 Hematology-Oncology Clinic, Center for Specific Organs Cancer, National Cancer Center, Goyang, 410-769, Korea; 4 Department of Hematology-Oncology, Ajou University School of Medicine, Suwon, 443-749, Korea

**Keywords:** peripheral T-cell lymphoma, not otherwise specified, lymphopenia, international prognostic index, prognostic factor

## Abstract

**Background:**

Peripheral T-cell lymphoma, not otherwise specified (PTCL-NOS) is a heterogeneous group of aggressive T-cell lymphomas with poor treatment outcomes. The aim of this study was to evaluate whether lymphopenia at diagnosis would have an adverse effect on survival in patients with PTCL-NOS treated with anthracycline-containing chemotherapy.

**Methods:**

A total of 118 patients with PTCL-NOS treated with anthracycline-containing chemotherapy from 4 Korean institutions were included.

**Results:**

Thirty-six patients (30.5%) had a low absolute lymphocyte count (ALC, < 1.0 × 10^9^/L) at diagnosis. Patients with lymphopenia had shorter overall survival (OS) and progression-free survival (PFS) rates compared with patients with high ALCs (*P *= 0.003, *P *= 0.012, respectively). In multivariate analysis, high-intermediate/high-risk International Prognostic Index (IPI) scores and lymphopenia were both associated with shorter OS and PFS. Treatment-related mortality was 25.0% in the low ALC group and 4.8% in the high ALC group (*P *= 0.003). In patients considered high-intermediate/high-risk based on IPI scores, lymphopenia was also associated with shorter OS and PFS (*P *= 0.002, *P *= 0.001, respectively).

**Conclusion:**

This study suggests that lymphopenia could be an independent prognostic marker to predict unfavorable OS and PFS in patients with PTCL-NOS treated with anthracycline-containing chemotherapy and can be used to further stratify high-risk patients using IPI scores.

## Background

Peripheral T-cell lymphomas (PTCL) account for approximately 12% to 15% of all non-Hodgkin's lymphomas in Western countries and 15% to 20% in Asian countries [1,2]. Peripheral T-cell lymphoma, not otherwise specified (PTCL-NOS), is the most common heterogeneous subgroup of PTCL because it includes lymphomas with no definitive clinical or biologic profile and it cannot be classified into a specific subtype [3]. PTCL-NOS is a highly aggressive lymphoma with a poor response to conventional chemotherapy and a 5-year overall survival (OS) of about 25% to 45% [4]. Anthracycline-containing chemotherapy, such as CHOP (cyclophosphamide, doxorubicin, vincristine and prednisone) or CHOP-like regimens, are considered to be standard therapy for PTCL-NOS, although remission rates are less than satisfactory [1]. More intensive regimens, such as hyper-CVAD (hyperfractionated cyclophosphamide, vincristine, doxorubicin, and dexamethasone) and hyper-CHOP, have not shown improved outcomes compared with CHOP regimens [5]. Several prognostic factors, including the International Prognostic Index (IPI), Prognostic Index for T-cell lymphoma (PIT), and International Peripheral T-cell Lymphoma Project (IPTCLP), have been suggested as methods to determine prognostic factors for outcomes with PTCL-NOS [6-9]. In addition, biologic markers such as nuclear factor (NF)-κB and cytochrome P4503A4 isoenzymes have been proposed; however, they do not stratify PTCL-NOS completely [3,10-12]. As a result, there is no single or simple clinical or biologic parameter for predicting treatment outcomes, except for IPI, in patients with PTCL-NOS. Because previous prognostic markers, such as IPI, have been based on information from all patients with PTCL-NOS regardless of chemotherapy regimen used, the role of IPI needs to be evaluated in patients treated with similar chemotherapy regimens. This would allow for identification of additional simple prognostic markers in the same population of patients.

Lymphopenia measured by absolute lymphocyte count (ALC) at diagnosis has been studied as an independent prognostic factor for poor survival in many hematologic malignancies, including Hodgkin's disease, diffuse large B-cell lymphoma (DLBCL), and follicular lymphoma [13-19]. In addition, it is known as a poor prognostic marker in solid tumors such as metastatic breast cancer and sarcomas [18]. Lymphopenia can also be used as a predictable marker for the relapse after chemotherapy; lymphocyte recovery after chemotherapy and autologous hematopoietic stem cell transplantation (ASCT) can help predict clinical outcomes in DLBCL patients [20,21]. A few studies have reported the clinical impact of lymphopenia in T-cell lymphoma. Recently, the role of lymphopenia at diagnosis was suggested as a powerful predictor of unfavorable treatment outcomes in extranodal natural killer/T-cell lymphoma (ENKL) [22]. Because there is no information on the role of lymphopenia at diagnosis of PTCL-NOS, we evaluated its prognostic value in the patients with PTCL-NOS treated with similar chemotherapy regimens. The objective of this study was to retrospectively investigate whether lymphopenia is a predictive marker for survival in patients with PTCL-NOS treated with anthracycline-containing chemotherapy.

## Patients and methods

### Patients

Patients diagnosed with PTCL between January 2000 and December 2009 from 4 Korean institutions were evaluated for inclusion into the study. Patients with a diagnosis of PTCL other than PTCL-NOS, such as anaplastic large cell lymphoma, angioimmunoblastic T-cell lymphoma, enteropathy-associated T-cell lymphoma, ENKL, subcutaneous panniculitis-like T-cell lymphoma, primary cutaneous T-cell lymphoma (e.g., mycosis fungoides) were excluded. Specific extranodal presentations of PTCL-NOS including primary central nervous system (CNS) lymphoma or primary cutaneous lymphoma were also excluded. Among 169 patients with PTCL-NOS screened, 21 were excluded for the following reasons: 2 patients for double primary cancer, 5 for up-front ASCT, 4 for primary CNS lymphoma, 5 for primary cutaneous lymphoma, and 5 for incomplete clinical data. Another 15 (8.9%) patients who did not receive chemotherapy because of poor performance status, combined comorbidity, or patient refusal were also excluded. A total of 133 patients received systemic chemotherapy. Of these, 118 (88.7%) patients were treated with anthracycline-containing chemotherapy (e.g., CHOP or CHOP-like regimens) as first-line treatment and 15 (11.3%) were treated without anthracycline-containing chemotherapy (e.g., IMEP [ifosfamide, etoposide, methotrexate, prednisone]). Therefore, 118 patients were included in the trial.

Medical records were retrospectively reviewed for patient demographics. These included age (< 60 vs. ≥ 60 years), gender (male vs. female), Eastern Cooperative Oncology Group (ECOG) performance status (0-1 vs. 2-4), the presence of B symptom (present vs. absent), Ann Arbor stage (1-2 vs. 3-4), the number of extranodal sites involved (0-1 vs. ≥ 2), bone marrow involvement (positive vs. negative), lactic dehydrogenase (LDH) concentrations (normal vs. elevated), ALC (≥ 1.0 × 10^9^/L vs. < 1.0 × 10^9^/L), and prognostic scores such as IPI (low risk/low-intermediate risk vs. high-intermediate/high risk) and PIT (group 1-2 vs. group 3-4). For this study, lymphopenia was defined as an ALC less than 1.0 × 10^9^/L. Complete blood counts (CBC) with differential and chemistry were performed at the time of diagnosis and prior to treatment. No patients showed clinical signs of severe infection at the time of laboratory testing. The study protocol was approved by the institutional review board from each participating institution.

### Prognostic scores

IPI scores were based on age, ECOG performance status, LDH concentrations, the number of extranodal sites involved, and Ann Arbor stage as described above [9]. Four risk groups were defined by IPI score: 0 to 1, low risk; 2, low-intermediate risk; 3, high-intermediate risk; and 4 to 5, high risk. PIT scores were calculated using age, ECOG performance status, LDH, and bone marrow involvement as described above. Four risk groups were defined by PIT scores: 0, group 1; 1, group 2; 2, group 3; and 3 to 4, group 4 [6].

### Treatment and response

Anthracycline-containing chemotherapy included CHOP (n = 98), CHOP-like regimens (n = 14), ProMACE/CytaBOM (prednisone, cyclophosphamide, doxorubicin, etoposide, cytarabine, bleomycin, vincristine, and methotrexate; n = 2), CAVOP (cyclophosphamide, doxorubicin, etoposide, vincristine, and prednisolone; n = 2), or hyper-CVAD (n = 2). Tumor response was defined as a complete response (CR), partial response (PR), stable disease, and progressive disease according to the International Workshop criteria [23]. Overall response rate (ORR) was defined as the proportion of patients achieving a PR or better.

### Statistical methods

The significance for categorical variables was calculated using the chi-square test. Continuous variables were compared by the t-test. Overall survival (OS) was measured from the first date of diagnosis until death from any cause, with surviving patients censored at the last follow-up date. Progression-free survival (PFS) was defined as the time from the date of diagnosis until disease progression, relapse after response, or death due to lymphoma or treatment. Death from other causes or survival at last follow-up were censored. Survival curves were plotted by the Kaplan-Meier method and compared using the log-rank test. The influence of each prognostic factor identified by univariate analysis was assessed by multivariate analysis using Cox proportional-hazards regression stepwise method. A *P*-value < 0.05 was considered statistically significant for all analyses. All statistical analyses were performed using SPSS for Windows, Version 18.0.

## Results

### Patient characteristics

A total of 118 patients were treated with anthracycline-containing chemotherapy. The study group consisted of 79 males (66.9%) and 39 females (33.0%) with a median age of 56 years (range, 20-86 years). Fourteen (11.8%) patients presented with a poor performance status, and 32 (27.1%) had B symptoms at diagnosis. Seventy-nine (66.9%) patients had stage III or IV advanced disease. The number of patients with extranodal involvement at more than 1 sites and involvement of bone marrow were 35 (29.6%) and 33 (27.9%), respectively. Sixty-one (51.6%) patients had elevated LDH. For IPI scores, 44 (37.3%) patients were classified as low risk, 31 (26.3%) as low-intermediate risk, 30 (25.4%) as high-intermediate risk, and 13 (11.0%) as high risk. For PIT scores, 30 (25.4%) patients were classified in group 1, 43 (36.4%) in group 2, 28 (23.7%) in group 3, and 17 (14.4%) in group 4.

### Clinical characteristics according to absolute lymphocyte count

The median ALC was 1.32 × 10^9^/L (range, 0.039-5.03 × 10^9^/L). Patients were divided into 2 groups according to ALC (≥ or < 1.0 × 10^9^/L). The proportion of patients with a low ALC was 30.5% (36 of 118 patients). For patients classified as having a high ALC (n = 82), the median level was 1.78 × 10^9^/L (range, 1.04-5.03 × 10^9^/L). Patients with low ALC (n = 36) had a median level of 0.69 × 10^9^/L (range, 0.039-0.98 × 10^9^/L). The clinical characteristics of patients according to ALC are shown in Table 1. The following characteristics were similar between the 2 groups: age, gender, performance status, presence of B symptom, Ann Arbor stage, number of extranodal sites involved, and involvement of bone marrow. The average number of cycles of first-line chemotherapy given was lower in low ALC group compared with the high ALC group (*P *= 0.007). Elevated LDH, high-intermediate/high risk IPI scores and high PIT scores were correlated with a low ALC (*P *= 0.031, *P *= 0.043, *P *= 0.010, respectively).

**Table 1:** Patient characteristics according to absolute lymphocyte count

	High ALC (*N *= 82)	Low ALC (*N *= 36)	*P*-value
Age			
< 60 vs. ≥ 60 years	51/31	21/15	0.692
Gender			
Male vs. Female	55/27	24/12	0.966
Performance status			
0-1 vs. 2-4	74/8	30/6	0.355
B symptom			
Present vs. Absent	21/61	11/25	0.578
Stage			
1-2 vs. 3-4	31/51	8/28	0.098
Extranodal involvement			
0-1 vs. ≥ 2	62/20	21/15	0.059
Bone marrow involvement			
Positive vs. Negative	21/61	12/24	0.389
LDH			
Normal vs. Elevated	45/37	12/24	0.031
IPI			
L, LI vs. HI, H	57/25	18/18	0.043
PIT			
Group 1-2 vs. 3-4	57/25	16/20	0.010
CR			
CR vs. non-CR	42/37	14/12	0.952
Response (≥ PR)			
Responder vs. Non-responder	61/18	17/9	0.231
TRM during the 1^st ^line chemotherapy			
Yes vs. No	4/78	9/27	0.003
Cycles of 1^st ^line Chemotherapy			
Median, range	6 (1-8)	3 (1-9)	0.007

LDH, lactate dehydrogenase; IPI, International Prognostic Index; L, low; LI, low-intermediate; HI, high-intermediate; H, high; PIT, Prognostic Index for peripheral T-cell lymphoma; CR, complete response; PR, partial response; TRM, treatment related mortality; ALC, absolute lymphocyte count.

### Response according to absolute lymphocyte count

Among the 118 patients who were treated with anthracycline-containing chemotherapy, 105 were evaluable for treatment response. Fifty-six (47.4%) patients achieved a CR and 78 (66.1%) achieved a PR or better. The CR rate was 53.2% (42 of 79 patients) and the ORR was 78.2% (61 of 79 patients) in the high ALC group. For the low ALC group, the CR rate was 53.8% (14 of 26 patients) and the ORR was 65.4% (17 of 26 patients). There were no statistically significant differences in the CR rate and ORR based on ALC (Table 1).

### Overall survival and progression-free survival analysis

The median duration of follow-up was 27.6 months (range, 1.0-69.2 months). Sixty (50.8%) patients died during the follow-up period. The rate of treatment-related mortality (TRM) during first-line anthracycline-containing chemotherapy was 11.0% (13 of 118 patients); 25.0% (9 of 36 patients) in low ALC group and 4.8% (4 of 82 patients) in high ALC group (*P *= 0.003). The 3-year estimate for OS was 48.5% and PFS was 35.0%.

**Figure 1:**
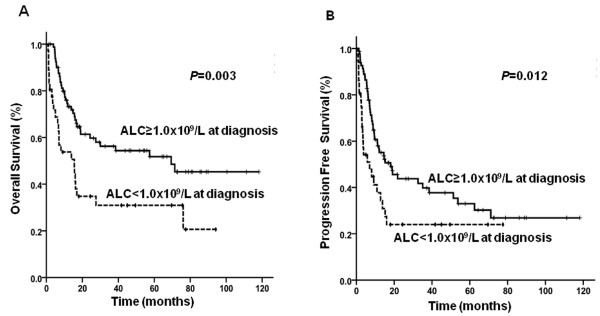
**Overall survival (A, *P *= 0.003) and progression free survival (B, *P *= 0.012) according to absolute lymphocyte count (ALC)**.

The median OS was longer in patients with high ALCs compared to those with low ALCs (69.4 months vs. 15.5 months, *P *= 0.003; Figure 1A). In univariate analysis, the following variables were associated with an unfavorable OS: poor performance status (*P *< 0.001), number of extranodal sites involved ≥ 2 (*P *= 0.005), elevated LDH (*P *< 0.001), high-intermediate/high risk IPI (*P *< 0.001), and PIT groups 3, 4 (*P *< 0.001; Table 2). In multivariate analysis, IPI (hazard ratio [HR] 4.06, 95% CI 2.40-6.84, *P *< 0.001) and lymphopenia (HR 2.24, 95% CI 1.33-3.78, *P *= 0.002) were independent prognostic factors for predicting OS in patients with PTCL-NOS (Table 3).

**Table 2:** Univariate analysis for overall survival and progression free survival in patients with PTCL-NOS

	Median OS, months	HR (95% CI)	*P*-value	Median PFS, months	HR (95% CI)	*P*-value
Age						
< 60 years	69.4	1.54 (0.92-2.58)	0.100	11.9	1.00 (0.62-1.62)	0.976
≥ 60 years	18.9			13.8		
Gender						
Male	30.0	1.24 (0.73-2.11)	0.422	12.1.	0.91 (0.55-1.51)	0.732
Female	27.5			15.2		
Performance status						
0-1	57.4	4.09 (2.10-8.00)	< 0.001	14.5	2.28 (1.16-4.46)	0.016
2-4	6.3			3.6		
B symptom						
Absent	38.5	1.45 (0.84-2.50)	0.178	14.7	1.31 (0.79-2.16)	0.282
Present	15.6			7.1		
Stage						
1-2	69.4	1.63 (0.93-2.87)	0.086	19.1	1.68 (1.02-2.77)	0.041
3-4	16.6			9.5		
Extranodal involvement						
0-1	69.4	2.10 (1.24-3.54)	0.005	18.1	2.08 (1.29-3.35)	0.003
≥ 2	11.5			8.1		
Bone marrow involvement						
Negative	57.4	1.62 (0.93-2.79)	0.084	14.5	1.68 (1.02-2.76)	0.039
Positive	10.2			7.3		
LDH						
Normal	NR	2.89 (1.67-5.00)	< 0.001	16.0	1.69 (1.06-2.68)	0.025
Elevated	11.5			8.8		
IPI						
L, LI	NR	3.96 (2.36-6.66)	< 0.001	18.1	2.34 (1.47-3.74)	< 0.001
HI, H	8.1			8.1		
PIT						
Group 1-2	76.1	2.78 (1.67-4.62)	< 0.001	15.2	1.69 (1.06-2.69)	0.026
Group 3-4	10.1			9.4		
ALC						
≥ 1.0 × 10^9^/l	69.4	2.19 (1.30-3.67)	0.003	18.1	3.01 (1.14-3.02)	0.012
< 1.0 × 10^9^/l	15.5			7.0		

LDH, lactate dehydrogenase; IPI, International Prognostic Index; L, low; LI, low-intermediate; HI, high-intermediate; H, high; PIT, Prognostic Index for peripheral T-cell lymphoma; ALC, absolute lymphocyte count; NR, not reached; OS, overall survival; PFS, progression free survival.

**Table 3:** Multivariate analysis for overall survival and progression free survival in patients with PTCL- NOS

	OS *P*-value	HR (95% CI)	PFS *P*-value	HR (95% CI)
IPI				
L, LI	< 0.001	4.06 (95% CI 2.40-6.84)	< 0.001	2.43 (95% CI 1.51-3.90)
HI, H				
ALC				
≥ 1.0 × 10^9^/l	0.002	2.24 (95% CI 1.33-3.78)	0.008	1.94 (95% CI 1.19-3.18)
< 1.0 × 10^9^/l				

IPI, International Prognostic Index; L, low; LI, low-intermediate; HI, high-intermediate; H, high; ALC, absolute lymphocyte count; OS, overall survival; PFS, progression free survival.

The median PFS was longer in patients with high ALCs compared to those with low ALCs (18.1 months vs. 7.0 months, *P *= 0.012; Figure 1B). Poor performance status (*P *= 0.016), advanced stage (*P *= 0.041), number of extranodal sites involved ≥ 2 (*P *= 0.003), bone marrow involvement (*P *= 0.039), elevated LDH (*P *= 0.025), high IPI scores (*P *< 0.001), and high PIT scores (*P *= 0.026) were associated with a shorter PFS by univariate analysis. Of these factors, high IPI scores (HR 2.43, 95% CI 1.51-3.90, *P *< 0.001) and lymphopenia (HR 1.94, 95% CI 1.19-3.18, *P *= 0.008) were significant independent prognostic factors for predicting PFS by multivariate analysis (Table 3).

### Survival analysis of high-intermediate/high-risk IPI patients

**Figure 2:**
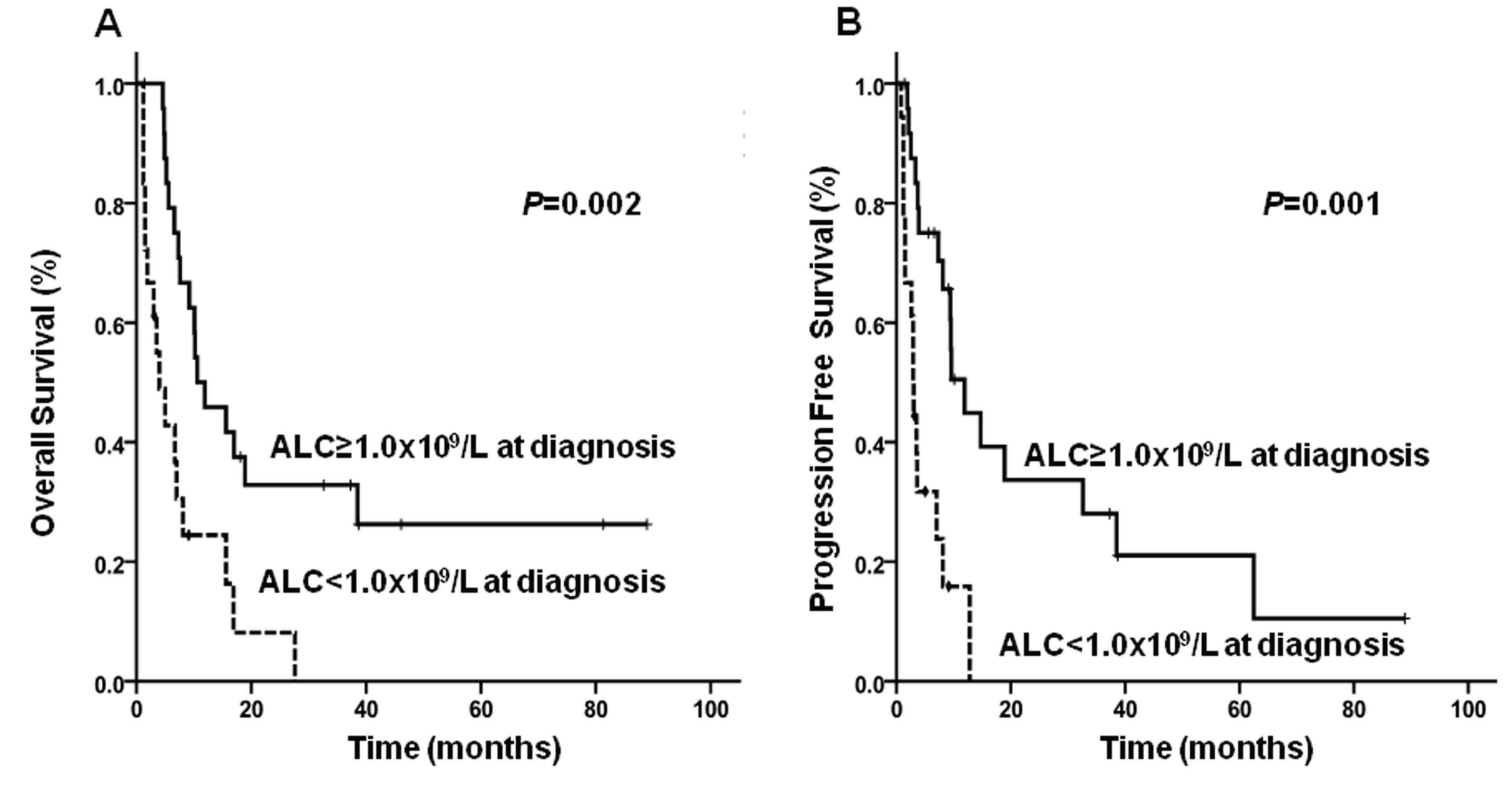
**Overall survival (A, *P *= 0.002) and progression free survival (B, *P *= 0.001) according to absolute lymphocyte count (ALC) in PTCL, NOS patients with high-intermediate/high risk IPI**.

Forty-three (36.4%) patients were categorized as high-intermediate/high risk by IPI scores. The median follow-up duration was 8.1 months (range, 4.3-11.8 months). Eighteen (41.9%) patients were in the low ALC group. Thirty-three patients (76.7%) died during the follow-up period. The TRM rate during first-line anthracycline-containing chemotherapy was 4.0% (1 of 25 patients) in the high ALC group and 38.8% (7 of 18 patients) in the low ALC group (*P *= 0.006). The 3-year estimate for OS was 20.1% and for PFS was 17.9%. The median OS was longer in patients in the high ALC group--10.6 months (range, 3.9-17.2 months) versus 4.0 months (range, 1.1-6.8 months) in the low ALC group (*P *= 0.002). Lymphopenia was also independently associated with an unfavorable impact on OS (HR 3.09, 95% CI 1.52-6.32, *P *= 0.002) and PFS (HR 4.01, 95% CI 1.80-9.00, *P *= 0.001; Figures 2A and 2B). No other variables were significantly associated with OS or PFS by univariate analysis (Table 4).

**Table 4:** Univariate analysis for overall survival and progression free survival in high-intermediate and high risk IPI patients

	Median OS, months	HR (95% CI)	*P*-value	Median PFS, months	HR (95% CI)	*P*-value
Age						
< 60 years	7.0	0.79 (0.39-1.61)	0.512	2.9	0.50 (0.24-1.01)	0.059
≥ 60 years	9.2			9.6		
Gender						
Male	10.1	1.33 (0.64-2.76)	0.444	8.1	1.07 (0.29-2.29)	0.862
Female	5.2			3.5		
Performance status						
0-1	10.1	1.72 (0.79-3.73)	0.168	8.1	1.15 (0.51-2.59)	0.728
2-4	4.8			3.6		
B symptom						
Absent	9.2	1.29 (0.62-2.66)	0.490	8.1	1.09 (0.51-2.33)	0.810
Present	7.0			3.6		
Stage						
1-2	4.0	0.21(0.02-0.72)	0.147	3.6	0.45 (0.05-3.44)	0.445
3-4	9.2			8.1		
Extranodal involvement						
0-1	10.1	1.13 (0.56-2.28)	0.729	14.7	1.27 (0.61-2.67)	0.518
≥ 2	8.1			3.9		
Bone marrow involvement						
Negative	11.9	1.33 (0.66-2.67)	0.414	9.5	1.29 (0.64-2.61)	0.467
Positive	7.3			7.0		
LDH						
Normal	9.2	1.51 (0.45-4.97)	0.498	8.1	1.54 (0.46-5.10)	0.474
Elevated	7.6			7.3		
PIT						
Group 1-2	16.9	1.35 (0.58-3.14)	0.476	8.1	1.06 (0.45-2.48)	0.883
Group 3-4	7.6			7.3		
ALC						
≥ 1.0 × 10^9^/l	10.6	3.09 (1.52-6.32)	0.002	11.9	4.02 (1.80-8.97)	0.001
< 1.0 × 10^9^/l	4.0			2.9		

LDH, lactate dehydrogenase; IPI, International Prognostic Index; L, low; LI, low-intermediate; HI, high-intermediate; H, high; PIT, Prognostic Index for peripheral T-cell lymphoma; ALC, absolute lymphocyte count; OS, overall survival; PFS, progression free survival.

## Discussion

This study found that lymphopenia is an unfavorable prognostic factor for patients with PTCL-NOS treated with anthracycline-containing chemotherapy. Higher IPI scores and lymphopenia prior to chemotherapy were independent prognostic factors for shorter OS and PFS in these patients. Lymphopenia was frequently observed in patients with elevated LDH, high-intermediate/high risk IPI scores, and high PIT scores (*P *= 0.031, *P *= 0.043, *P *= 0.010, respectively). However, lymphopenia had a significant role in identifying subgroups with a poorer prognosis among patients at high-risk according to IPI scores; in these patients, lymphopenia was associated with significantly shorter OS and PFS (*P *= 0.003, *P *= 0.012, respectively).

Both IPI and PIT scores have been used as important prognostic factors in PTCL [6,9]. Recently, IPI scores were found to be the most reliable factor in predicting survival, but PIT scores had no significant prognostic role according to the IPTCLP [24]. However, the prognostic value of both these factors were studied regardless of the type of systemic chemotherapy. Moreover, no parameters were used to predict treatment outcomes or further stratify patients with the same IPI scores. Castillo et al. reported that a PIT score > 2 and lymphopenia were independent prognostic factors for predicting a poor response to therapy and survival in 69 patients with PTCL-NOS [25]. However, this study included only 37 patients who were treated with systemic chemotherapy [25]. Therefore, there was insufficient evidence to determine the prognostic role of lymphopenia in PTCL-NOS. According to our data, 53.3% (8 of 15) of untreated patients did not receive chemotherapy because of poor performance status, and 60.0% (9 of 15) of these patients had lymphopenia at diagnosis. Poor performance status and lymphopenia might be frequently observed in patients who do not receive chemotherapy. Therefore, any analysis of the prognostic role of lymphopenia should be performed only among patients who receive similar systemic chemotherapy. In our study, we enrolled patients newly diagnosed with PTCL-NOS who were treated with anthracycline-containing chemotherapy as first-line treatment and excluded patients who did not receive any treatment or who received up-front ASCT. Therefore, patients enrolled in our study may be more homogenous compared with patients from previous studies and may be more appropriate for evaluating prognostic factors.

The causes for lymphopenia are multi-factorial and its consequences are heterogeneous. First of all, lymphopenia could be related to inflammation, a condition usually accompanied by relative neutrophilia or absolute lymphopenia. In certain situations, inflammatory mediators seem to play an important role in the development and progression of cancers [26]. There are some reports on the relationship between inflammation and cancer treatment outcomes via the transcription factors NF-κB in PTCL [11,27]. In our study, lymphopenia was not simply a result of impairment of bone marrow function, because there was no significant difference in bone marrow involvement between ALC groups (*P *= 0.578). It could be suggested that lymphopenia may be related to higher tumor burden and increased inflammatory mediators because we found that lymphopenia was closely related to elevated LDH, high-intermediate/high risk IPI scores, and high PIT scores. However, there may be other clinical meanings of lymphopenia besides tumor burden, since lymphopenia was an independent prognostic factor even among patients classified as high-intermediate/high risk based on IPI scores.

ALC is a surrogate marker of host immunity. Because lymphopenia is reflective of a damaged immune system, patients with lymphopenia usually showed poor response and survival rates [25]. Previous studies have explained why low ALCs might be related to immune suppression or be a consequence of lymphocytic cytokines produced by lymphoma cells [18]. Plonquet et al. reported that a low NK cell count was related to a poor response to chemotherapy in patients with DLBCL treated with rituximab [28]. CD4 lymphopenia is known to be an independent risk factor for febrile neutropenia and early death in cancer patients receiving cytotoxic chemotherapy [29]. Therefore, lymphopenia may increase a patient's vulnerability to infection during chemotherapy. Infection-related mortality is a main cause of death during the chemotherapy for lymphoma. In our study, TRM during first-line anthracycline-containing chemotherapy was significantly higher in patients with low ALCs compared to those with high ALCs (25.0% vs. 4.8%, *P = *0.003), even though there was no difference in the treatment response rates between the 2 groups (*P *= 0.154). Therefore, survival differences according to the ALC are not associated with a poor response to chemotherapy, but rather to a high rate of early mortality during the chemotherapy. In conclusion, chemotherapy regimens should be carefully selected for patients with PTCL-NOS and lymphopenia in order to reduce TRM during first-line chemotherapy. Newly developed targeted agents or cellular therapy for treatment of these patients should be considered in the future.

Furthermore, lymphocyte analysis at the time of diagnosis could clarify the role of lymphopenia in PTCL-NOS. Although IPI scores showed a significant role for predicting survival, ALC--a simple and easily obtainable test--was also found to have an independent role in predicting survival of patients with PTCL-NOS. Therefore, a lymphocyte count should be recommended as a standard test before initiation of first-line chemotherapy for PTCL-NOS.

Our study has some limitations. Because this study was conducted retrospectively, treatment regimens were not identical. To overcome this problem, we enrolled a relatively large number of patients newly diagnosed with PTCL-NOS who were treated with anthracycline-containing chemotherapy as first-line treatment. In this regard, large-scale, prospective studies are required to confirm the prognostic value of lymphopenia compared to other biologic tests, such as immunophenotyping or gene expression profiling. In addition, we did not perform a review of the pathology of each case, since cases had already been reviewed by experienced hematopathologists from each institution.

In conclusion, we found that lymphopenia was an independent prognostic factor for poor OS and PFS in patients with PTCL-NOS treated with anthracycline-containing chemotherapy. Lymphopenia was also a useful marker for further stratification of patients at high risk based on IPI scores. Further efforts to reduce TRM and new strategies to improve OS are needed, especially in patients with PTCL-NOS and lymphopenia.

## Competing interests

The authors declare that they have no competing interests.

## Authors' contributions

YRK involved in conception, design, data interpretation, and manuscript writing. JSK performed data interpretation and revising it critically for intellectual content. SJK involved in acquisition of data, analysis of data. HAJ involved in acquisition of data, analysis of data. SJK involved in acquisition of data, analysis of data and participating in comprehensive discussion. WSK involved in analysis of data and participating in comprehensive discussion. HWL involved in acquisition of data, analysis of data. HSE involved in acquisition of data, analysis of data and participating in comprehensive discussion. SHJ involved in acquisition of data, analysis of data and participating in comprehensive discussion. JSP involved in acquisition of data, analysis of data and participating in comprehensive discussion. JWC involved analysis of data and participating in comprehensive discussion. YHM involved in analysis of data and participating in comprehensive discussion. All authors read and approved the final manuscript.
